# Current Status of Polysaccharides-Based Drug Delivery Systems for Nervous Tissue Injuries Repair

**DOI:** 10.3390/pharmaceutics15020400

**Published:** 2023-01-25

**Authors:** Caterina Valentino, Barbara Vigani, Giuseppina Sandri, Franca Ferrari, Silvia Rossi

**Affiliations:** Department of Drug Sciences, University of Pavia, Via Taramelli 12, 27100 Pavia, Italy

**Keywords:** polysaccharides, drug delivery systems, spinal cord injury, peripheral nerve injury

## Abstract

Neurological disorders affecting both CNS and PNS still represent one of the most critical and challenging pathologies, therefore many researchers have been focusing on this field in recent decades. Spinal cord injury (SCI) and peripheral nerve injury (PNI) are severely disabling diseases leading to dramatic and, in most cases, irreversible sensory, motor, and autonomic impairments. The challenging pathophysiologic consequences involved in SCI and PNI are demanding the development of more effective therapeutic strategies since, as yet, a therapeutic strategy that can effectively lead to a complete recovery from such pathologies is not available. Drug delivery systems (DDSs) based on polysaccharides have been receiving more and more attention for a wide range of applications, due to their outstanding physical-chemical properties. This review aims at providing an overview of the most studied polysaccharides used for the development of DDSs intended for the repair and regeneration of a damaged nervous system, with particular attention to spinal cord and peripheral nerve injury treatments. In particular, DDSs based on chitosan and their association with alginate, dextran, agarose, cellulose, and gellan were thoroughly revised.

## 1. Introduction

The nervous system is the apparatus of the human body that functions to connect the various structures of the organism, but also to react to external stimuli [1]. It is composed of two major regions: The central nervous system (CNS) and the peripheral nervous system (PNS). The first is the most complex apparatus of the human body and includes the brain and its caudal prolongation, the spinal cord [2,3]. The CNS is connected to the periphery of the body by an extensive network of nerves composing the PNS [4]. The PNS includes the neural tissue outside the CNS, such as paravertebral and neuro-vegetative ganglia, peripheral nerves that extend from the brain and the spinal cord (cranial and spinal nerves, respectively), and specific sensory organs [5]. Neurological disorders affecting both CNS and PNS still represent one of the most critical and challenging pathologies, therefore many researchers have been focusing on such field in the last decades [6].

**Figure 1:**
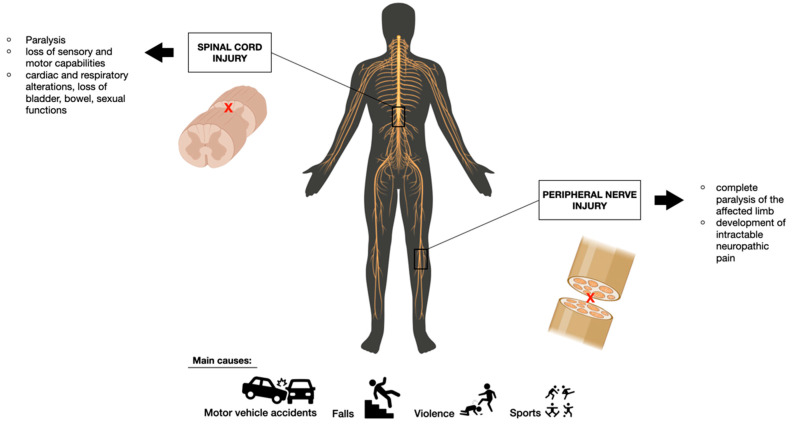
Spinal cord injury and peripheral cord injury: Main causes and symptoms.

Spinal cord injury (SCI) and peripheral nerve injury (PNI) are severely disabling diseases leading to dramatic sensory, motor, and autonomic function impairments. Mechanical trauma causing compression of the spinal cord is, generally, the main and most common cause of SCI, which can be the result of motor vehicle accidents, falls, sports-related injuries, or violence [7]. The consequence of such compression results in a cascade of events including cellular, biochemical, and vascular events, exacerbating the damaged area and promoting the formation of a glial scar, responsible for the establishment of physical and chemical barriers to whatever endeavor to stimulate axonal regeneration [6,8]. On the other hand, PNI is defined as the loss of structure and/or function of peripheral nerves, characterized by permanent disablement and severe motor function defects, which could lead to the complete paralysis of the affected limb or the development of intractable neuropathic pain, with a severe impact on a patient’s lifestyle [9,10]. Such pathology can be caused by accidents, traumatic conditions, surgery, other events such as ischemia, and chemical or thermal causes [9,11]. Main causes and symptoms of SCI and PNI are represented in Figure 1.

Generally, PNS injuries are more common than those of the CNS, due to the lack of protection provided by the blood–brain barrier and the braincase [12]. Notably, in contrast to CNS, peripheral nerve fibers exhibit a remarkable intrinsic potential for self-regeneration. Schwann cells (SCs) are the main cells responsible for the activation of a cell-intrinsic myelin breakdown process through autophagy. Since myelin is the first responsible for storing substances (known as myelin-associated glycoproteins) that prevent the regeneration of damaged axons, this mechanism is acknowledged as the primary mechanism of the regenerative potential after PNI. [13,14]. Nevertheless, the achievement of total regeneration is generally unsatisfactory, providing weak motor recovery and irreversible sensory dysfunction [9]. In fact, an endogenous spontaneous repair of the damaged peripheral nerve is possible only for small gaps (<5 mm); it is partial and, in most cases, accompanied by a reduction in sensory and motor functions [11].

The challenging pathophysiologic consequences involved in SCI and PNI demand the development of more effective therapeutic strategies since, as yet, a therapeutic protocol that can effectively lead to a complete recovery from such pathologies is not available. Nowadays, the therapeutic approach of SCI is mostly limited to providing supportive relief to patients and is focused on the modulation of secondary complications, which hinder SCI treatment after the damage, and the stimulation of functional recovery via rehabilitation [15,16]. Currently, the most common treatments involve neurorrhaphy and allo- and autologous nerve grafting, which represents gold-standard treatments for nerve gaps smaller or higher than 1 cm, respectively [17,18]. These techniques have been, however, overcome due to their various critical issues and limitations, namely, excessive stretching of the nervous tissue and an impairment of nerve vascularization, in the case of neurorrhaphy; on the other hand, nerve grafting is often compromised by the scarce availability of nerve grafts, the morbidity of the donor site, the immunological responses, and the possible neuroma formation [17,19].

The development of tissue-engineered grafts represents a promising strategy for the treatment of lesions of the nervous system; in the case of PNI, such systems are generally named neural guide conduits (NGCs) [19]. The versatility of the geometries of NGCs and the possibility of loading various therapeutic substances (including drugs, cells, and/or growth factors) allow these products to be used in a combined neuroprotective and neuroregenerative approach in order to promote neuronal recovery [20,21,22].

Polysaccharides are natural polymers consisting of repeated mono- or disaccharide units linked via glycosidic bonds, which are generally isolated from several sources, such as plants, terrestrial and marine animals, or microorganisms [23]. Drug delivery systems (DDSs) composed of polysaccharides have been receiving more and more attention for a wide range of applications, due to their outstanding physical-chemical properties [24]. Due to the possibility of being neutral or presenting positive or negative charges, the linear or branched molecular structure and their wide range of molecular weights (from a few hundred to several thousand Daltons) are some of their attractive physical-chemical properties. Moreover, polysaccharides are recognized for their biocompatibility, bioactivity, biodegradability, and low immunogenicity [23,25,26,27].

**Figure 2:**
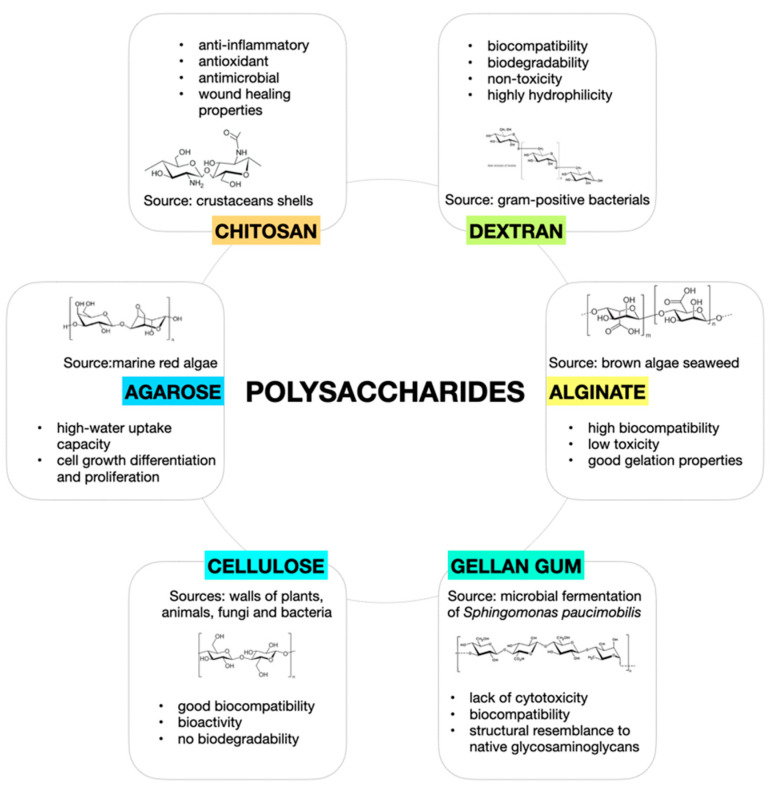
Graphical description of main polysaccharides, their principal sources, and properties.

Figure 2 reports a graphical description of the main polysaccharides and their principal sources and properties.

Due to the above-mentioned characteristics, polysaccharides can be regarded as functional excipients for the setup of effective DDSs for the treatment of nervous tissue injuries [24,28].

This review aims to provide an overview of the most studied polysaccharides used for the development of DDSs intended for the repair and regeneration of a damaged nervous system, with particular attention to spinal cord and peripheral nerve injury treatments. In particular, DDSs based on chitosan and their association with alginate and hyaluronic acid, alginate, dextran, agarose, cellulose, and gellan were thoroughly revised.

## 2. Chitosan (CS)

Chitosan (CS) is a promising polysaccharide consisting of N-acetyl-D-glucosamine and D-glucosamine units linked by 1-4-β-glycosidic bonds, which is generally obtained from the deacetylation of chitin, derived from crustacean shells [29]. This polysaccharide has the unique properties of being polycationic and is characterized by a multitude of features, such as anti-inflammatory, antioxidant, antimicrobial, and wound-healing properties [30]. CS has been widely used for neural tissue engineering, due to its physical, chemical, and mechanical properties, and especially for its similarity to glycosaminoglycans forming the extracellular matrix [31].

As for SCI treatment, both hydrogels and nano-scale DDSs endowed with antioxidant and/or anti-inflammatory properties able to fill the gap generated by the injury and control the release of bioactive molecules were proposed in the last decade.

The first evidence of CS application for SCI treatment was reported by Skop et al., who developed genipin-crosslinked CS-based microspheres, produced via the coaxial airflow technique and intended for the delivery of cells and growth factors for nervous tissue regeneration. CS was ionically bound with heparin, a well-known anionic glycosaminoglycan with anticoagulant and anti-inflammation properties, as well as a high affinity for growth factors. The system was characterized by high biocompatibility towards the neural stem cell line and easy binding to the fibroblast growth factor, which is an important factor for neural stem cell survival [32].

Wu and coworkers developed nanoparticles (Nps) with neuroprotective potential, based on the chemical interaction between glycol CS, a water-soluble CS derivative, and ferulic acid (FA). Glycol CS and FA can form hydrophobically self-assembled Nps consisting of a hydrophilic shell (glycol CS) and a hydrophobic core (FA). The neuroprotective effect of Nps was assessed by a glutamate-induced excitotoxicity model on primary spinal cord neuron culture. Moreover, Nps were injected in a rat spinal cord contusion injury model to assess the bioavailability, pharmacokinetics, and functional recovery after Nps systemic administration. Axons and neuron cells at the lesion site were greatly recovered by the delivery of FA-glycol CS Nps, and the number of activated astrocytes and macrophages was reduced. These neuroprotective benefits ultimately contributed to SCI functional recovery [33].

Another CS–drug complex was investigated by Gwak et al., who prepared CS Nps via an ionotropic gelation process, based on the amide-coupling method, to provide intracellular delivery of methylprednisolone (MD), a cortico-steroid used for the treatment of acute SCI as an anti-inflammatory agent and to reduce neurological deficits after injury. In addition to drug delivery, the authors aimed at investigating possible gene delivery due to the positive charges of chitosan, which can bind with the negative charges of DNA through electrostatic interaction. Nps determined low cytotoxicity on mouse neural stem cells, and plasmid DNA was efficiently delivered once Nps were injected in vivo in a compressed spinal cord injury model, producing effective protein expression. Inflammation and apoptosis were reduced even at a low MD dose [34].

Ni and coworkers explored the sustained delivery of chondroitinase ABC (ChABC), an enzyme able to decompose glycosaminoglycans chains of chondroitin sulfate proteoglycans (CSPGs), which are recognized for their negative impact on axon regeneration after SCI, and which, therefore, are able to elicit an indirect positive effect on axonal regeneration. For this purpose, ChABC-loaded CS microparticles were prepared via ionotropic gelation, using tripolyphosphate (TPP) as cross-linking agent. Solid microparticles were then mixed with polypropylene carbonate and subjected to electrospinning to obtain microfibers containing CS-ChABC microspheres. The systems were able to assure a stable and prolonged release of ChABC in vitro. Moreover, fibers containing microspheres were implanted in vivo in a hemisected thoracic spinal cord with improved axonal regeneration and animal functional recovery [35].

Wang and collaborators investigated the release of valproic acid from CS Nps. Valproic acid was found to be neuroprotective for microglia and reduce inflammation induced by nervous tissue injury. After intravenous injection in a rat SCI model, Nps enhanced the functional recovery of nervous tissue and inhibited astrocytes activity and, thus, inflammation. Furthermore, the disruption of the blood spinal cord barrier occurring after SCI was recovered, and neuroprotection was successfully achieved [36].

Alizadeh et al. developed a thermo-sensitive hydrogel based on CS for the delivery of the nerve growth factor (NGF). The gel consisted of CS, β-glycerol phosphate disodium salt pentahydrate as a gelling agent and hydroxyethylcellulose as a cross-linking agent; the hydrogel solutions were prepared at 4 °C. The hydrogel was used as a vehicle for the delivery of lentiviral-mediated NGF-overexpressing human adipose-derived mesenchymal stem cells (hADSCs), which are recognized to improve neural growth and neural regeneration due to the secretion of neurotrophic and neurovascular factors and are able to differentiate into Schwann-like cells. Both transduced hADSCs alone and in combination with the CS-based hydrogel were injected in vivo in a contusive spinal cord injury model one week after surgery, and the hydrogel effectiveness was evaluated two months after surgery. The combination of transduced hADSCs and the hydrogel was more effective than transduced hADSCs alone in both repairing SCI and providing the functional recovery of animals [37].

A CS-collagen-based hydrogel loaded with a serine protease inhibitor, serpine (Serp-1), which is an immune-modulating biologic drug, was developed for the treatment of crush-induced SCI by Kwiecien and collaborators. Both low (10 μg) and high doses (100 μg) of Serp-1 were loaded into CS-collagen hydrogels, which were then injected in a dorsal column crush SCI rat model. Locomotor functionality was assessed and histopathologic analysis was performed; the authors proved that hydrogel loaded with the highest Serp-1 dose reduced the damaged area, resulting in better and faster motor recovery and diminished neurological deficits, in comparison with the low-dose-loaded and pristine hydrogel. Furthermore, a reduction of neural injury was observed with the high-dose-loaded hydrogel, which was attributed to a Serp-1-induced reduction of apoptosis [38].

Wang and coworkers designed stearic acid (SA)-CS nanomicelles loaded with sesamol, a polyphenol with strong antioxidant properties but low cellular uptake and biocompatibility and, therefore, requiring a drug carrier system for suitable administration. CS nanomicelles conjugated with SA were developed via centrifugation followed by freeze-drying to obtain a solid product. Nanomicelles proved to be stable in phosphate buffer solution (PBS) for 15 days and able to provide a sustained release of sesamol, reaching almost 100% after 50 h from the beginning of the in vitro release experiment. In vitro tests were performed on NSC-34 cells, a hybrid motoneuron-like cell line; cells were treated with lipopolysaccharide (LPS) before being incubated with the samples (nanomicelles or free sesamol), in order to investigate nanomicelles’ protective effect. Other in vitro tests were carried out on the same cell line (MTT, LDH, and intracellular ROS assay). The best results were always observed for loaded nanomicelles, confirming their neuroprotective potential and antioxidant properties. Moreover, loaded nanomicelles were also able to modulate the levels of apoptotic genes and reduce the expression of inflammation-related genes [39].

A CS-based hydrogel was prepared by Javdani et al. by adding a sodium hydroxide solution to a CS solution; the hydrogel was then loaded with Selenium-loaded Nps. Selenium was selected for its relevance as a nutrient for humans and animals and for its fundamental role in many metabolic pathways, namely for its beneficial effect on acute SCI. In vivo studies were performed on rat models: Animals were subjected to aneurysm clamping at the level of the thoracic vertebrae to induce SCI, and the effect of both free and loaded hydrogels was compared with that of the control group (no drug intervention). Histological evaluation of the group treated with the loaded hydrogel highlighted a reduction in the severity of bleeding and the number of inflammatory cells, as well as the occurrence of new nerve fibers. The presence of selenium within the hydrogel was responsible for the inhibition of the cellular pathways related to inflammation and was demonstrated to provide neuronal recovery and protection. Hence, the selenium-loaded CS hydrogel showed potential antioxidant and anti-inflammatory properties for the treatment of SCI [40].

**Figure 3:**
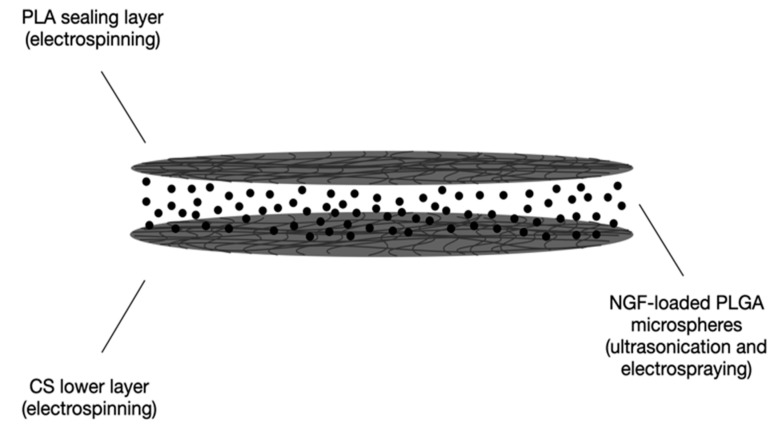
Schematic representation of the sandwich-structured drug delivery composite scaffold developed by Song and coworkers [41].

Song and collaborators developed a CS scaffold intended for the SCI treatment. A sandwich-structured composite system for long-lasting controlled release of NGF (2 months, required by the healing process of SCI) was prepared via electrospray and electrospinning techniques. A poly(lactic acid) (PLA) film was used as a sealing layer to impair drug diffusion outside the system and provide mechanical support; the sandwich layer consisted of poly(lactic-co-glycolic acid) PLGA microspheres loaded with NGF, and a CS film constituted the lower layer to host bone marrow mesenchymal stem cells (BMSCs). The PLA and CS films were prepared by electrospinning whilst the NGF-loaded PLGA microspheres were obtained via ultrasonication. In detail, the final composite system was obtained by electrospraying NGF-loaded PLGA microspheres above the PLA fibrous membrane and subsequently electrospinning the CS solution above the previous two layers. A schematic representation of the composite scaffold is displayed in Figure 3.

In vitro drug release measurements demonstrated that the composite system enables a prolonged release of NGF for more than 2 months. The biocompatibility of the system was tested in vitro on a PC-12 cell line for 7 days; neurite outgrowth from PC-12 cells was promoted by the composite system. BMSCs were then seeded onto the CS film. When implanted in a rat model, the final system was found to promote and improve neuroregeneration and locomotor functional recovery of SCI within 8 weeks of evaluation [41].

In addition to the treatment of SCI, researchers have focused their efforts on developing CS-based DDSs for the treatment of PNI. In this context, CS employment as an NGC component seemed particularly promising, due to the physical and chemical structure similarity of these systems with the multi-layer 3D architecture of peripheral nerves. CS is also capable of supporting axonal regrowth, ameliorating functional recovery after injury, healing, and reducing scar formation [31,42].

Regarding PNI, a variety of systems of various types, including NGCs, hydrogels, and Nps loaded with various active molecules with immunosuppressant, neuroprotective, and nerve regeneration enhancement properties, were developed during the last decade.

Li and collaborators developed a CS guide loaded with FK506, an FDA-approved immunosuppressant agent generally used to avoid allograft rejection after transplantation and considered for its neuroprotective and neurotrophic potential. A tubular scaffold was obtained by solvent casting into a specific mold presenting an inner stainless-steel tube and an outer tube, and subsequent soaking in a NaOH solution to obtain a tubular gel. These guides were implanted in vivo on a sciatic nerve injury rat model, and after 6–8 weeks, electrophysiological and histological analyses were performed. The FK506 loaded-CS guide decreased the inflammatory reaction and allowed faster reinnervation, in comparison with the groups treated with a silica guide or CS guide without FK506 [43].

A CS conduit was designed by Farahpour and Ghayour. It was loaded with Acetyl-L-carnitine (ALC), a natural amino acid derivative with both neuroprotective and antinociceptive effects recognized for its potential in improving nerve regeneration after PNI. A CS solution was prepared at 50 °C, and the addition of glycerol was exploited to improve CS mechanical properties, excluding fragility. The CS conduit was prepared by injecting a CS/glycerol solution in a homemade mold with an internal diameter of 1.8 mm and an external one of 2 mm. As for in vivo efficacy studies, animals (rats) were divided into four groups: (i) The transected control group, (ii) the sham-surgery group, (iii) the group treated with CS alone, and (iv) the group treated with ALC-loaded CS conduit. The last group demonstrated improved functional recovery of the transected sciatic nerve, with the promotion of both motor and sensory regeneration and reinnervation of the injury [44].

**Figure 4:**
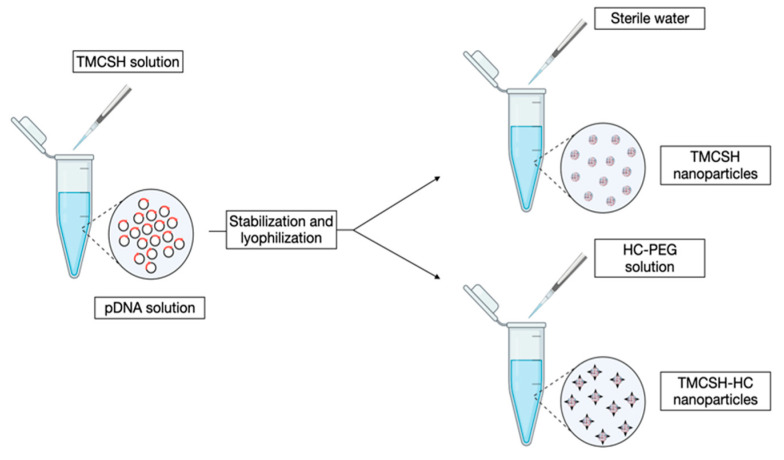
Schematic representation of TMCSH-based nanoparticle preparation, inspired by Lopes and collaborators [45].

CS Nps were employed to obtain a sustained gene release for peripheral nerve regeneration after intramuscular administration, which is clinically relevant and not considered invasive. In detail, a thiolated trimethyl CS (TMCSH) was in contact with pDNA encoding for the brain-derived neurotrophic factor (BDNF) for 15 min, which is well-known for its protective action towards neuron survival after injury. BDNF-loaded Nps were prepared according to the method reported in Figure 4; a plasmid encoding for tetanus neurotoxin (HC), which can modulate nanoparticle retrograde transport after peripheral administration, was added as the HC-PEG solution to the TMCSH solution to obtain TMSCH-HC Nps. For in vivo studies, rats were injected with Nps 8 days before nerve crush injury to investigate the effect of a therapeutic agent on the prevention of axon degeneration and/or nerve regeneration. After in vivo administration, Nps proved to stimulate injury recovery, avoid nerve degeneration, and improve nerve regeneration [45].

A CS-based guide conduit was conceived by Manoukian et al. for the sustained release of 4-Aminopyridine (4AP), a potassium-channel blocker able to accelerate nerve innervation. The nerve guidance conduit was characterized by the presence of aligned microchannels obtained through unidirectional freezing, achieved by pouring a CS solution into a homemade mold that was then submersed in liquid N_2_; subsequent freeze-drying of the CS solution allowed the attainment of a highly porous scaffold with a foam-like structure. Halloysite nanotubes (HNTs) loaded with 4AP were also added to the CS solution to improve the strength of the final system and provide a prolonged release of the drug, controlling the initial burst effect. The final porous system was cross-linked using alkaline epichlorohydrin. The authors observed an in vitro drug release from the 4AP-HNTs-loaded CS conduit of almost 98% within 7 days, differently from the 4AP-HNTs and 4AP- load CS matrix, which both showed faster release. The cross-linked 4AP-HNTs-loaded CS conduit displayed the longest sustained release profile (almost 30% at 7 days). In vitro studies carried out on SCs proved the positive effect of 4AP as an enhancer of the upregulation of NGF or BDNF, which are key trophic factors for axon regeneration and nerve remyelination after injury. A sciatic nerve defect rat model was exploited for in vivo preliminary assessment, which corroborated the biocompatibility of the scaffold and the infiltration of SCs within the lumen of the scaffold, following the organized structure of the aligned channels [22].

CS Nps loaded with curcumin and containing SCs cells were developed by Jahromi et al. and loaded into poly-L-lactide acid (PLLA)-based multi-wall carbon nanotube conduits. Curcumin has been demonstrated to decrease SCs apoptosis and enhance the number of myelinated axons inside the injury. Therefore, the authors investigated curcumin association with SCs with the purpose of improving axons regeneration. Specifically, a hollow PLLA/multi-wall carbon nanotube conduit (prepared by electrospinning apparatus equipped with a rotating rod) was filled with a fibrin-based hydrogel containing curcumin-encapsulated CS Nps and SCs. The final scaffold showed good biocompatibility and the ability to promote SCs adhesion, mostly when the PLLA/multi-wall carbon nanotube conduit was at the lowest concentration. A sciatic nerve injury rat model was employed to evaluate the scaffold ability of damage recovery; in particular, the composite system was demonstrated to boost nerve regeneration and improve locomotor functionality when compared to the autograft used as control [46].

Zeng et al. developed CS/PLGA microspheres for the delivery of NGF; microspheres were prepared by a re-emulsification TPP ionic cross-linking method. The release of NGF from microspheres was between 65 and 45% on the 49th day, depending on the TPP concentration used to cross-link the microspheres; upon increasing TPP concentration, a higher reduction in drug release rate in comparison with lower TPP concentration was observed. Microspheres demonstrated their ability to promote neurite formation when tested on PC12 cells thanks to NGF binding to the tyrosine kinase receptor, which activates intracellular pathways that induce neurite extension. In vivo experiments on the sciatic nerve rat model highlighted the ability of axons to regenerate the loaded microspheres [47].

### 2.1. CS Associations

Chitosan has also been employed in association with other polysaccharides to develop DDSs useful for both SCI and PNI repair and regeneration. Chitosan’s association with other polysaccharides is linked to its cation charges, which enable ionic interaction with anionic polysaccharides, such as hyaluronic acid (HA) and alginate (ALG) [48,49,50,51]. The association with other polysaccharides can be useful not only to enhance mechanical and biomimetic properties of the systems developed, but also to better control the drug release from these systems [48,52].

#### 2.1.1. CS/HA Association

In the context of SCI regeneration, HA and glycol CS were employed in association to develop a hydrogel loaded with tauroursodeoxycholic acid (TUDCA), a bile acid with cytoprotective activity and anti-neuroinflammatory properties. In detail, oxidized hyaluronate and glycol CS solutions were mixed in different ratios and then cross-linked with TUDCA. The gel obtained was subjected to freeze-drying, and a porous lyophilized product with regular pore size was obtained. The cross-linked gel was tested in vivo in a mechanical SCI model on rats and demonstrated its ability to promote functional recovery by reducing the expression of pro-inflammatory cytokines and thus inhibiting inflammatory pathways involved in SCI [50].

As for PNI treatment, the complementary advantages of CS and HA hydrogel were investigated by Zhang and collaborators, who developed an injectable hydrogel for the prolonged delivery of NGF for peripheral nerve regeneration. An injectable CS/HA 1:1 hydrogel was prepared at 37 °C, using ethyl-3-(3-dimethylaminopropyl) carbodiimide (EDC) and N-hydroxysuccinimide (NHS) to promote an amide reaction between components. The gelation time, measured by a tilt test, was very fast (3 min) at pH 7.4. The freeze-dried hydrogel was characterized by a highly porous interconnected structure with macropores, with the cross-section desirable to enhance axon and nerve cell growth and the absorption of bioactive substances for nerve regeneration. NGF release in PBS shows an initial rapid release due to the diffusion of NGF and the swelling of the hydrogel in an aqueous medium; then a slower release was observed, reaching approximately 80% of the loaded drug in 56 days. The loaded hydrogel demonstrated the highest biocompatibility towards Bone Marrow Mesenchymal Stromal Cells (BMMSCs) when compared to the controls (blank group without NGF, pure components, and NGF-free hydrogel). Moreover, freeze-dried loaded hydrogel determined high cell adhesion and promoted their differentiation [48].

#### 2.1.2. CS/ALG Association

Chitosan has also been used in association with ALG by Vigani and coworkers for the delivery of a sigma 1 receptor agonist, named RC-33, intended for the treatment of SCI. RC-33 is a promising active molecule able to promote neurite outgrowth in PC12 cells induced by the nerve growth factor. Thus, the study was carried out to develop a scaffold characterized by both neuroregenerative potential due to the natural polysaccharide-based scaffold and neuroprotective action due to the presence of the loaded active molecule. RC-33 was incorporated into ALG electrospun nanofibers due to ionic bonds formed between the anionic polysaccharide and the cationic drug candidate; loaded fibers were then subjected to cross-linking with calcium chloride to obtain a water-insoluble product. Cross-linked nanofibers were subsequently loaded into a CS film produced via solvent casting to obtain a flexible and easy-to-handle final product. Good cell biocompatibility of the scaffold was evidenced in human neuroblastoma SH-SY5Y cells [51].

The association of CS with ALG was also investigated by Rahmati and collaborators to develop a hydrogel for the delivery of berberine (Ber) for the treatment of PNI, especially for sciatic nerve regeneration. Ber was employed because of its ability to improve peripheral nerve damage thanks to its antibacterial, immunostimulant, anticancer, and antimotility properties and beneficial effect on neurological disorders. An ALG solution was cross-linked with CaCl_2_ and added dropwise to a CS solution containing β-glycerol phosphate as a cross-linking agent. Different concentrations (0.1, 1, and 10% *w*/*w*) of Ber were then added to the ALG/CS solution. Loaded freeze-dried hydrogels displayed a highly interconnected porous structure, capable of providing a constant Ber release for 24 days. Hemocompatibility of the hydrogels was demonstrated on human anticoagulated blood collected from volunteers, and their biocompatibility was confirmed on PC12 cells. In vivo studies on a crush-induced sciatic nerve rat model demonstrated a positive effect, especially for the 1% Ber loaded-hydrogel, indicating that Ber has potential activity in nerve regeneration [52].

Table 1 presents a summary of all the CS-based DDSs discussed in the text.

## 3. ALGINATE

ALG is a natural linear anionic polysaccharide, generally extracted from brown algae seaweed, consisting of repeated units of (1-4)-β-d-mannuronic acid and an α-l-guluronic acid building block [53]. ALG possesses several fruitful features, encompassing high biocompatibility, low toxicity, and good gelation properties, which make it an ideal polymer for the development of a scaffold intended for tissue engineering and drug delivery, due to its interaction with bivalent cations. Namely, ALG has been widely used in the field of nervous tissue engineering to develop various novel DDSs: Injectable or non-injectable hydrogels, microfibers, and more complex composite systems with both neuroprotective and neuroregenerative potential [13,54].

ALG was exploited by Downing and coworkers in 2012 to produce a microfibrous drug delivery system containing rolipram, an anti-inflammatory drug with many positive properties for the treatment of nerve damage and, in particular, SCI. The system developed was a poly(L-lactide)-based microfibrous platform produced via the electrospinning technique and coated with a CaCl_2_- cross-linked ALG hydrogel layer employed for the controlled and local delivery of rolipram. The developed platform confirmed its ability to control drug delivery, showing a burst release of approximately 40% rolipram within the first 18 h and a controlled release even after 1.5 days. The efficacy of low drug content in the platforms was pointed out after the treatment of rats subjected to a C5 hemisection lesion, with improvements in axon regeneration and functional and anatomical recovery [55].

Ansorena et al. developed an injectable ALG hydrogel embedded with microspheres loaded with the glial-derived neurotrophic factor (GDNF), a growth factor able to stimulate functional recovery by promoting survival and neurite growth. GDNF was encapsulated into polylactic-co-glycolic acid (PLGA) microspheres prepared via solvent extraction/evaporation by means of Total Recirculation One-Machine System (TROMS) technology to obtain a homogeneous product with a high encapsulation efficiency. ALG was mixed with fibrinogen to improve biocompatibility and cross-linked with calcium chloride (CaCl_2_) to obtain a hydrogel; prior to gelation, GNDF microspheres or free GNFD were added to the ALG:fibrinogen solution. The system was able to control the release of GNDF when encapsulated in PLGA microspheres and when in free form, but the release was slower in the presence of microspheres. A bioactivity assay of the released GNDF, performed on rat pheochromocytoma PC-12 cells, demonstrated the ability to promote cell differentiation in the presence of the growth factor, thanks to the development of neurites from cells. The performance of the ALG:fibrinogen hydrogel containing GNDF microspheres or free GNDF was tested in vivo on a rat model (the hydrogel was formed in situ via the direct injection of the components on hemisected spinal cords of rats); the ability of the GNDF-microsphere-loaded hydrogel to promote neurite formation around the lesion was proven; in contrast, the free GNDF-loaded hydrogel promoted neurite ingrowth at the lesion site. Finally, the functional recovery of the rats was then assessed, which showed the best overall recovery when treated with the free GNDF-loaded hydrogel, in comparison with non-treated animals and the GNDF-microspheres-loaded hydrogel; these results can be conceivably attributed to the different release profile of GNDF at the site of injury [56].

A similar injectable CaCl_2_ cross-linked ALG:-fibrinogen-based hydrogel was employed by des Rieux and coworkers as a carrier for the vascular endothelial growth factor (VEGF) for its neuroprotective properties in the spinal cord regeneration process. VEGF was used in its free form and encapsulated in CS–dextran sulfate (CS/Dx) Nps or in PLGA microspheres. The free VEGF loaded-hydrogel showed modest proliferation on human neuronal-like cells (SH-SY5Y cells) but not on mouse fibroblast-like cells (NIH-3T3 cells), even when supplemented with fibrinogen. Therefore, the free VEGF-loaded hydrogel stimulated neurite growth in ex vivo dorsal root ganglia cultures, but not when tested in a rat spinal cord hemisection model. VEGF Nps or microspheres were first characterized for the growth factor release profile; both systems provided a slower release when compared to the free VEGF-loaded hydrogel, which was too slow in the case of VEGF microspheres. Both free VEGF and VEGF Nps were then incorporated into the ALG:fibrinogen hydrogel to combine fast and sustained release, and the system was injected into a rat spinal cord hemisection model. VEGF was always demonstrated to stimulate angiogenesis in the lesion site and promote neurite growth in and around the lesion. However, the VEGF-loaded hydrogel was not able to improve the functional recovery of rats [57].

A drug delivery system intended to provide both neuroprotective and neuroregenerative effects was developed by Nazemi and collaborators; minocycline hydrochloride (MH) and paclitaxel (PCX) were chosen for their neuroprotective and neuro-regeneration activity, respectively. A dual system was prepared, composed of PLGA-based microspheres embedded in an ALG hydrogel; ALG sulfate (ALG-S) was also considered for its bioaffinity, due to electrostatic interactions. The synergistic effect of a formulation containing both MH and PCX-loaded PLGA microspheres was investigated. Regarding MH, an antibiotic and anti-inflammatory drug with potent neuroprotective activities, a complex with ALG or ALG-S was prepared, achieved via the formation of an electrostatic interaction and metal-ion chelation, by using CaCl_2_ or MgCl_2_ as cross-linking agents. The complex was obtained by mixing equal volumes of the drug solution with ALG or ALG-S solutions (drug and polymer at the same concentrations) and the drug–polymer interaction products were recovered after centrifugation and lyophilization. After UV-Vis spectroscopy analysis of the supernatants deriving from centrifugation, the MH ALG-S complex cross-linked with MgCl_2_ resulted in being the complex with the highest encapsulation efficiency. Three different hydrogel formulations were prepared by embedding either the free drug directly into the hydrogel or by its indirect incorporation when entrapped into the complex. In detail, two hydrogel formulations were obtained by directly mixing the free drug solution with either an ALG solution or an ALG:ALG-S solution at a ratio of 9:1, and a third hydrogel formulation was prepared via the indirect incorporation of the drug by embedding the MH ALG-S complex within an ALG solution. In all formulations, calcium D-gluconate monohydrate was used as a cross-linking agent to allow the formation of hydrogels. PCX was encapsulated in PLGA microspheres prepared via the single (oil/water) emulsion/solvent evaporation method. Loaded PLGA microspheres were added to the ALG:ALG-S solution and the hydrogel was obtained following the same protocol as for MH. An ALG: ALG-S hydrogel containing either PCX-loaded PLGA microspheres and MH was finally prepared. The final formulation containing both the drugs proved to be able to reduce the activity of inflammatory cells, thanks to the activity of MH, and to decrease the formation of fibrotic scars, as a result of the presence of PCX, when tested on a left lateral hemisection animal (rat) model of SCI. Moreover, an improvement in the functional recovery of animals was observed for the dual-drug delivery system, thus demonstrating its promising regeneration-enhancing properties toward nervous injuries [58].

**Figure 5:**
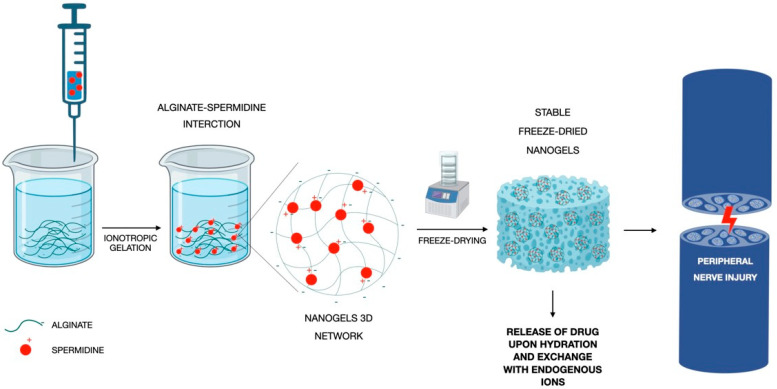
Graphical description of the development of SP-ALG cross-linked nanogels modified by Valentino et al. [59].

Recently, in a paper of ours, ALG was cross-linked with Spermidine (SP) to obtain nanogels as innovative tools for peripheral nerve repair. SP is a naturally occurring bioamine endowed with neuroprotective activity and a cationic nature, which allows its interaction with the anionic ALG. ALG at high and medium viscosity was cross-linked via the ionotropic gelation process with SP at different concentrations, and nano/microgels dispersions were obtained (Figure 5). A DoE approach was exploited in order to find the best combination of the two components in terms of the mean hydrodynamic diameter. Viscosity measurements and the solid-state characterization (FT-IR analysis) allowed us to confirm the occurrence of interactions between ALG and SP. The addition of trehalose as a cryoprotecting agent was also considered for the freeze-drying process, which was exploited to obtain a stable solid product. In vitro studies on SCs demonstrated the high biocompatibility of nanogels; antioxidant and anti-inflammatory properties of SP remained even after cross-linking with ALG, meaning that its incorporation in nanogels did not impair its bioactivity [59].

A summary of all the ALG-based DDSs is reported in Table 2.

## 4. DEXTRAN

Dextran (Dx) is a complex branched glucan characterized by α-1,6 glycosidic bonds between glucose monomers; it is generally obtained by several Gram-positive, facultatively anaerobe cocci such as *Leuconostoc* and *Streptococcus* strains [60]. It is a biocompatible, biodegradable, nontoxic, and highly hydrophilic polysaccharide that is extensively employed in medicinal products for its peripheral blood-flow-enhancing properties, namely the reduction of blood viscosity and the avoidance of blood clot formation, which prompt Dx use as antithrombolytic agent. Moreover, Dx has been widely used in nanomedicine and for drug delivery; its neutral charge makes it an ideal candidate for Nps synthesis, as it improves nonspecific cellular uptake [61].

As for the employment in nervous system injuries, Dx was used for the synthesis of DDSs intended for spinal cord damage repair by intravenous injection; Nps consisted of an association of ibuprofen and Dx for the delivery of methylprednisolone (MP). Ibuprofen and Dx were combined via esterification between the hydroxyl groups of Dx and the carboxylic acid groups of ibuprofen, activated with N, N-carbonyldiimidazole. System biocompatibility was demonstrated in vitro on BV-12 microglial cells. The blood plasma concentration of MP was further evaluated after intraperitoneal injection in an SCI rat model comparing MP-loaded NPs with a reference solution of MP; for all the times considered, the MP concentration from loaded NPs was higher than that of the reference solution, indicating NPs potential for longer drug circulation and better drug bioavailability. Locomotor functionality recovery and the rehabilitation of neurological deficits and nerve functions after SCI were also improved by ibuprofen-loaded NPs. These Nps were, moreover, found to be successful in the prevention of and the slowdown of neuronal regeneration, due to their anti-inflammatory effect [62].

Dx-loaded Nps were synthesized by Liu and collaborators for the delivery of PCX. Acetalated-Dx Nps were prepared for their reported neuroprotective activity by the microprecipitation method, finding a 1:5 PCX: acetalated-Dx ratio as the best for encapsulation efficiency, loading degree, and PCX release profile. Loaded Nps provided continuous PCX release for 7 days after injection at the site of injury, promoting neural regeneration, neuroprotection, and enhanced locomotor recovery in rats [63].

Acetalated-Dx was also employed by Li and coworkers to obtain microspheres loading Nps, intended for MP sustained release in the case of SCI. Nano-in-micro structured microspheres were prepared by means of a microfluidic flow-focusing device, to exploit a controlled in-droplet precipitation and thus ensure a high drug loading. The nano-in-micro particles obtained allowed a gradual release and higher stability of the loaded drug. Interestingly, the authors succeeded in preparing a system with a high mass fraction of MP, which is particularly desired after intrathecal administration in rats, thanks to the limited volume that can be administered. Such microspheres were injected in vivo after weight drop injury of the spinal cord and provided constant drug release, reduction of the damaged area, and the recovery of motor functionality 28 days after injury [64].

The three Dx DDSs are grouped in Table 3.

## 5. AGAROSE (AG)

AG is a water-soluble natural biocompatible polysaccharide extracted from marine red algae, consisting of repeating units of the disaccharide agarobiose, which, in turn, is composed of D-galactose and 3,6-anhydro-L-galactopyranose [65]. AG is characterized by the unique property of self-gelation at 37 °C, without the need for cross-linking agents, thus avoiding their eventual toxicity; such a polymer is also recognized for its high water-uptake capacity, which can promote cell growth, differentiation, and proliferation [66]. In addition, this marine polysaccharide exhibits switchable chemical reactivity for functionalization, strong bioactivity, remarkable mechanical properties, and strict similarity with the natural extracellular matrix (ECM). For these reasons, AG has gained special attention as a biomaterial for the setup of complex carriers for controlled DDSs. In particular, AG’s ease of cross-linking through physical interactions has encouraged its application in DDSs [67].

Hence, AG has been widely used for tissue engineering and drug delivery systems intended for several applications, such as cartilage and bone regeneration, brain and nervous defects, cardiovascular diseases, and skin wounds [65]. In the context of SCI and PNI, different systems have been investigated, especially in situ gelling systems or hydrogels with complex geometries, used as platforms to hold and host various other systems such as Nps and microtubes loaded with an active ingredient.

In situ gelling hydrogels for the repair of SCI and local delivery of BDNF were developed by Jain et al. in 2006. The ability of AG regarding in situ gelling and adapting to the shape of the nervous tissue injury was investigated. Since the optimal gelling temperature of AG is 17 °C, the authors developed a cooling system to cool AG in a few seconds when applied at the site of injury and assured its maintenance within the lesion without leaking the defect. BDNF was selected due to the need for neurotrophic factors for neuronal survival and axonal growth after trauma. Lipid microtubes loaded with BDNF were embedded within the AG scaffold. The formulation was able to determine a diffusive-based sustained release of the growth factors in 5–7 weeks and improve the ability of restored nervous fibers to enter the hydrogel thanks to BDNF chemo-attractive action. The scaffold was implanted in vivo in a dorsal over-hemisection rat model and proved to reduce the reactivity of the astrocytes and the production of chondroitin sulfate proteoglycans (CSPGs) due to the presence of BDNF. Moreover, 6 weeks after implantation, a minimal inflammatory response was observed [68].

In 2011, the same authors investigated the release of different bioactive molecules from the AG hydrogel scaffold embedded with lipid microtubes. The scaffold was loaded with, in addition to BDNF, the constitutively active (CA) cell division control protein 42 homolog (CA-Cdc42) and CA Rac Family Small GTPase 1 (CA-Rac1), namely Rho GTPases, which are responsible for the filopodial and lamellipodial extension of axonal growth cones after SCI. The study proved the sustained release of the loaded molecules for at least 2 weeks and the efficacy in vivo of the scaffold in terms of a reduction of astrocytes and CSPG deposition [69].

An AG hydrogel was also developed by Chvatal and coworkers to achieve a localized release of MD. In detail, MD was encapsulated in PLGA Nps produced via the double-emulsion method, and the loaded Nps were mixed with the AG hydrogel. The hydrogel–nanoparticle system was able to determine a slow release of the drug over 6 days. Moreover, such a system, once topically delivered in vivo in a spinal cord injury rat model, proved to promote the reduction of the injury volume and the decrease in secondary injury-related inflammation events within 7 days [70].

Lee and colleagues exploited AG for the preparation of lipid microtubes embedded in an AG-based hydrogel for the delivery of chondroitinase ABC (chABC), an enzyme able to digest CSPGs, which is responsible for the inhibition of axon growth after SCI. Before incorporation into lipid microtubes, chABC was stabilized with trehalose due to its thermal instability at 37 °C. The system was then injected in vivo in a spinal cord lesion rat model, where it determined a sustained delivery of chABC, which, in turn, maintained its ability to digest CSPGs for 2 weeks after injury. Axonal growth and functional recovery of the rat model employed, due to the action of thermally stabilized chABC, was also observed [71].

A complex AG scaffold consisting of microchannels organized in a honeycomb arrangement was developed by Gao and coworkers. In detail, the scaffold consisted of an AG platform characterized by inner micro (166 μm) multi-channel guides, deriving from fiber bundles consisting of hexagonally packed (honeycomb architecture) polystyrene fibers in a linear orientation. The system was loaded with syngeneic marrow stromal cells expressing BDNF, produced via a retroviral vector coding for full-length human BNDF. The authors proved that their bioengineered scaffold could support and guide axonal growth in a clinically relevant in vivo model, namely, the complete transection of a severe rat spinal cord injury [72].

Cox and coworkers developed PLGA Nps loaded with estrogen (E2), which is known for its anti-inflammatory, antioxidant, anti-apoptotic, and neurotrophic properties and its potential for neuroprotection in SCI. E2-loaded PLGA Nps, obtained via the nanoprecipitation method, were dispersed in an AG hydrogel and the efficacy of the scaffold was assessed in a moderate to severe SCI rat model. The E2 concentration in plasma was found to be twice that of the physiological one, and a wide cytokine profiling range was observed [73].

Shultz and collaborators developed an AG hydrogel for the local controlled delivery of physiological doses of thyroid hormone 3,3′,5-triiodothyronine (T3). The rationale is that the loss of oligodendrocytes occurring after SCI results in demyelination and a spared axons formation, which impair healing. Various molecules were compared in vitro, and the authors found that T3 was the most effective to promote oligodendrocytes differentiation. T3 must be necessarily delivered in situ due to its potential systemic side effects, therefore it was loaded onto an AG hydrogel as insoluble particles. A clinically appropriate unilateral cervical spinal cord contusion injury rat model was exploited and exhibited the ability of the scaffold to release a T3 dose consistent with safe doses for humans, the enhancement of oligodendrocytes differentiation, and remyelination after injury [74].

Wang et al. studied the neuroprotective activity of MH, which can target the secondary mechanism after injury, for the treatment of SCI. The drug was complexed with Dx sulfate by the metal ion-assisted interaction to achieve a sustained release; the complex was then loaded onto an injectable AG hydrogel in order to assure the localization of the system in the intrathecal space of the injury. The efficacy of the scaffold was assessed using a clinically relevant unilateral cervical contusion injury model in which the spinal cords of adult rats were impacted at the C5 level. The authors demonstrated that a high concentration of MH requested for SCI repair, not achievable through systemic administration but, however, safe for humans, can be employed if locally delivered [75].

Another research group explored the local delivery of BDNF from an AG hydrogel with the aim of solving the diaphragmatic respiratory impairment attributed to cervical SCI. AG hydrogel was used as a host scaffold for Dx-CS-BDNF particles, self-assembled by electrostatic interactions. The final complex, once injected into the intrathecal space at the injury site in a unilateral cervical contusion rat model, demonstrated its potential efficacy for the effective restoration of respiratory functions [76].

Similarly, Gao and coworkers developed a multi-channel guidance scaffold for the regeneration of PNI. The developed system loaded with BDNF was the same as the previous work reported [72], but in this case, the microchannels had a diameter of 200 μm. After implantation in 15 mm long rat sciatic nerve gaps, the scaffold successfully guided linear axons regeneration over long distances, due to the system architecture and BDNF release at the distal and proximal nerve ending [77].

Table 4 reports all the DDSs based on AG for SCI applications.

## 6. CELLULOSE (CL)

Cellulose (CL) is the most abundant biopolymer found in the cell walls of plants or produced by animals, fungi, and bacteria. Structurally, it is primarily composed of D-glucopyranose ring units linked by β-1, 4-glycosidic linkages and organized in chains forming fibrillary units, which are, in turn, assembled in microfibrils [78]. CL has been widely employed for many pharmaceutical purposes due to its tunable properties, in terms of chemical, physical, and mechanical cues; moreover, it is characterized by biocompatibility and bioactivity, although it is not biodegradable [79]. This last feature could be useful for long-term applications, such as the case of nerve tissue recovery after injuries. Bacterial CL has been widely used for nervous tissue damage regeneration, as reported by Jabbari et al., 2022 [80]. For instance, biosynthesized cellulose (BC) obtained from *Gluconacetobacter hansenii* was employed by Stumpf and coworkers to prepare nerve guides able to enhance nerve regeneration after SCI. Authors prepared BC tubes (BCTs) containing various BC amounts depending on *G. hansenii* cells’ cultivation at different times (3, 6, 9, 12, 16, 18, and 22 days). BCTs were characterized by an inner diameter of 3.53 mm, a length of 5 cm, and mechanical properties similar to those of native neuronal tissue. BCTs were loaded with NGF as a neurite stimulation agent. NGF-loaded BCTs demonstrated good mechanical properties; in particular, BCTs after 22 days of cultivation (BCTs22) showed Young’s Modulus similar to that of the spinal cord. BCTs22 enabled an NGF-controlled release for 7 days, with an initial (8 h) burst release, due to the release of NGF molecules from the outer tube surface. Furthermore, NGF maintained its bioactivity on PC12 cells after release from BC tubes [81].

CL in association with a soy protein isolate was employed by Luo et al. for the development of tubes with an inner diameter of 1.5 mm and an outer diameter of 1.8 mm, intended for the repair of PNI. The soy protein isolate was used for its high biocompatibility, biodegradability, and processability. Tubes were seeded with SCs and pyrroloquinolinequinone (PQQ), a neurotrophic factor able to enhance SCs proliferation and migration. In vivo studies on a rat model were carried out. An autograft nerve, retained as the ‘gold standard’, was employed as a positive control. It highlighted the ability of the scaffold, especially when seeded with both cells and PQQ, to enhance nerve regeneration, even if to a minor extent in comparison with the control [82].

Table 5 summarizes all the DDSs based on CL described here.

## 7. GELLAN GUM (GG)

Gellan gum (GG) is a linear anionic exopolysaccharide, produced by microbial fermentation of *Sphingomonas paucimobilis*, composed of tetrasaccharide (1,3-β-D-glucose, 1,4- β-D-glucuronic acid, 1,4-β-D-glucose, and 1,4-α-l-rhamnose (Rha) repeating units with one carboxyl lateral group [83]. More specifically, the structure of GG is characterized by repeating tetramers of L-rhamnose, D-glucuronic acid, and two D-glucose subunits. GG is ductile, thermo-responsive, and can withstand acid and heat stress [84]. The most appealing properties of GG that qualify it as a suitable material for tissue engineering and DDS development are the lack of cytotoxicity, biocompatibility, structural resemblance to native glycosaminoglycans, and mechanical properties comparable to the elastic moduli of common tissues [85]. These properties make it an interesting material for the setup of various DDSs, including particles, films, fibers, and hydrogels. The pH-dependent swelling and stability of GG are desirable properties for DDS formulation, as well as its anionic nature that enables its gelation when combined with monovalent or divalent cations [86].

GG has been widely exploited for nerve tissue regeneration and, as reported in this section, both nanofibers and hydrogels were prepared and loaded with active molecules with positive charges for the treatment of both SCI and PNI.

**Figure 6:**
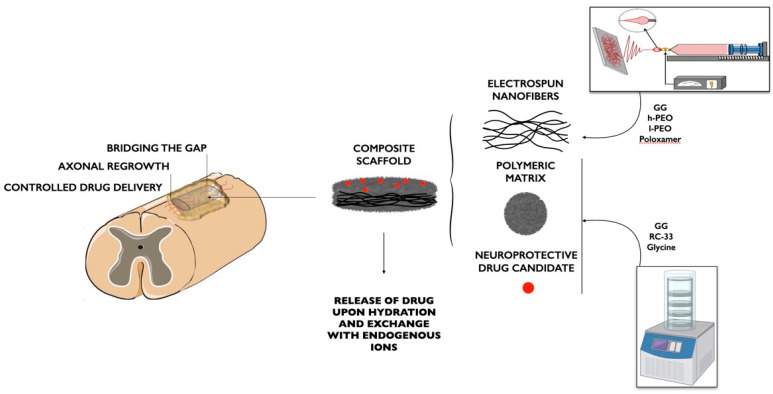
Illustration of the composite scaffold composed of GG electrospun fibers embedded within a GG porous matrix containing RC-33 drug candidate [87].

As for SCI treatment, GG was employed by Vigani and coworkers to develop a composite system for the delivery of RC-33, a drug candidate already used by the same authors, which cross-links with anionic polymers due to its positive charge, as illustrated in Figure 6 [51]. CaCl_2_-cross-linked GG nanofibers were prepared by the electrospinning process via the addition of two grades of poly (ethylene oxide) (PEO) (high (h-PEO) and low (l-PEO) molecular weight) and poloxamer (P407) to enhance GG electrospinnability. Electrospun nanofibers were embedded within an outer freeze-dried matrix, composed of an RC-33/GG interaction product and glycine as a cryoprotectant agent, where different concentrations of the drug candidate were loaded. The RC-33/GG interaction product was investigated by dialysis equilibrium, and the maximum binding capacity of GG for RC-33 was found. Four different loaded matrices, which provided the outer structure of the composite system, were obtained varying the concentration of RC-33, based on the results of dialysis equilibrium analysis. It was found that the matrix loaded with the highest amount of RC-33 (interacting with 40% of GG binding sites) was the optimal candidate in terms of mechanical and hydration properties. Finally, cross-linked nanofibers were immersed in a 40%RC-33/GG solution and freeze-dried to obtain the final composite system, characterized by improved mechanical properties [87].

**Figure 7:**
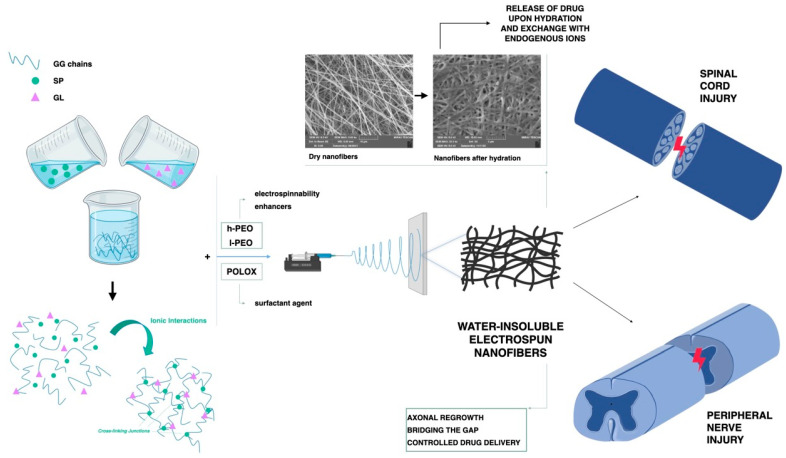
Representative scheme of preparation of GG-based nanofibers containing gelatin and SP developed by Vigani and coworkers for nervous tissue injuries [88].

The same authors used GG to prepare nanofibers loaded with SP and gelatin (GL), intended for both SCI and PNI treatment. Due to its positive charges, SP was used both as a cross-linking agent and as the active component to be released with a positive effect in the treatment of peripheral nerve regeneration. Mixtures containing GG and increasing SP concentrations (0–0.125% *w*/*w*) were prepared to investigate GG/SP interaction. The addition of GL was found to enhance system biomimetic properties. The best mixture of GG/SP/GL was electrospun to obtain cross-linked nanofibers (Figure 7). These fibers were characterized for mechanical properties and morphology after soaking in water for 24 h, thus confirming the formation of an interaction product since nanofibers were insoluble in an aqueous medium. Nanofiber biocompatibility was evaluated on SCs, showing that the presence of GL was mandatory to enhance nanofibers’ compatibility with cells [88].

Regarding PNI treatment, porous neurodurable hydrogel conduits made of GG and xanthan gum intercalated with polymethyl methacrylate (PMMA) particles were developed by Ramburrun and collaborators, for the controlled release of two model compounds: Bovine serum albumin (BSA) and diclofenac sodium. Hydrogel conduits were synthesized via a thermal-ionic cross-linking mechanism with direct addition via the intercalation of PMMA. Fifteen different formulations, chosen on the basis of a Box–Behnken experimental design, were evaluated for drug release, swelling, degradation, and textural properties. All the formulations gave an almost zero-order release of BSA and diclofenac sodium over the course of 20 and 30 days, respectively. This result was obtained due to the combination of a pH-responsive (pH 7.4) dissolution of the PMMA particles and the distinct gelling and degradation properties due to the presence of xanthan gum. The variation of the gellan–xanthan ratio and the pore-inducing properties of intercalated PMMA enabled fine-tuning of the mechanical properties of the hydrogel matrices, particularly the matrix rigidity and flexibility [89].

Li and coworkers developed a GG hydrogel loaded with laminin and NGF to promote neuronal stem cells’ proliferation and differentiation. Laminin is recognized as one of the most important elements of ECM of peripheral nerve cells and is responsible for the enhancement of neurite outgrowth. In detail, thiolated GG hydrogel was obtained by dissolving GG at 60 °C and, and after cooling at room temperature, the gel was mixed with a cell suspension containing laminin and NGF. NGF release was studied by immersing the hydrogel in PBS at 37 °C and analyzing the collected solution with a Sandwich ELISA test. The release was rather fast (approximately 60% in 20 h) and the release profile of NGF corresponded to a Fickian diffusion-controlled mechanism. In vitro experiments were carried out with neural stem cells harvested by the cerebral cortex of rats; cell viability after contact with the loaded GG hydrogel was manually determined after 72 h via a cell-counting kit (CCK-8), and cell proliferation was measured by fluorescence microscopy. Neural stem cells were found to be highly interconnected to the hydrogel, creating a whole network with an oval or triangular architecture [90].

Table 6 summarizes all the GG DDSs developed during recent years of research.

## 8. Conclusions

The success of DDSs in SCI and PNI treatment depends on different factors: The bioactive properties of the constituent polymers; the architecture and mechanical properties of the DDS that should mimic the natural extracellular matrix to support cell growth and assembly; and the efficacy of the loaded drug and the kinetics of drug release from the DDS.

Polysaccharides are the first choice of polymers since they are characterized by excellent biocompatibility with intrinsic biological cues and excellent intrinsic properties that facilitate their use in the fabrication of 3D structures able to mimic ECM and control the release of the loaded drug.

Moreover, since these polymers are generally derived from renewable sources or are byproducts of industrial processes, their use is to be considered collaborative in waste management and promising for an improved sustainable circular economy.

The revision of the literature reported here demonstrated how active the research in this field is. In recent years, many polysaccharide-based DDSs have been developed and have been proven effective in the treatment of nerve tissue injuries in animal models. Most of them are complex systems obtained by the assembly of different architectures (such as nanoparticles, fibers, films, and hydrogels) that require the employment of different techniques and, thus, a multistep process for their fabrication. Making the manufacturing process as simple as feasible, removing the crucial processes, and favoring its scalability are issues that will need to be addressed in the upcoming years.

Therefore, future research should be focused on the development of bioactive polysaccharide-based DDSs able to control the release of the loaded drug according to the therapeutic needs and characterized by a simple and scalable manufacturing process. Among the formulations reported, micro/nanofibers and micro/nanoparticles obtained by electrospinning and spray-drying, respectively, that are simple, versatile, highly efficient, and easily scalable using continuous manufacturing technologies are those that fulfill this requirement. Another issue of concern is the sterilization process employed. In many of the papers reviewed, no mention of this aspect is reported. The complexity of the systems developed could make it difficult to find a suitable method. Moreover, it must be underlined that no information about the translatability in humans of the DDSs reviewed here is present in the literature. The lack of in vivo tests on humans could arise as a problem, as mentioned above.

## Figures and Tables

**Table 1:** Summary of CS- and CS-association-based DDSs for SCI and PNI application. DDS description, production technique, therapeutic agent delivered, and in vitro and in vivo models exploited for efficacy assessment are reported.

DDS	Production Technique	Therapeutic Agent	Application	In Vitro Model	In Vivo Model	Reference
**CS microspheres**	Genepin-cross-linking and coaxial airflow technique	Heparin	SCI	Neural stem cell line	-	[32]
**Glycol/CS Nps**	Self-assembling	Ferulic acid	SCI	Primary spinal cord neurons culture	Rat spinal cord contusion injury model	[33]
**CS Nps**	Ionotropic gelation	Methylprednisolone	SCI	Mouse neural stem cells	Compressed spinal cord injury rat model	[34]
**Microfibers containinig** **ChABC-loaded CS microparticles**	Ionotropic gelation (with TPP)and electrospinning	Chondroitinase ABC	SCI	-	Hemisected thoracic rad spinal cord model	[35]
**CS Nps**	conjugating by coupling carboxyl to amino group in the presence of modification reagents and dyalization to isolate the conjugates	Valproic acid	SCI	-	Spinal cord contusion rat injury model	[36]
**CS-based thermo-sensitive hydrogel**	Hydroxyethylcellulose as cross-linking agent, β-glycerol phosphate disodium salt pentahydrate as gelling agent for CS solution	Lentiviral mediated NGF–overexpressing hADSCs	SCI	-	Contusive rat spinal cord injury model	[37]
**CS-collagen based hydrogel loaded with Serp-1**	Lyophilization	Serpine (Serp-1)	SCI	-	Dorsal column crush rat model	[38]
**CS-stearic acid conjugated nanomicelles loaded with sesamol**	Centrifugation followed by freeze-drying	Sesamol	SCI	NSC-34 cell line	-	[39]
**CS Hydrogel loaded with Nps**	Addition of sodium hydroxide	Selenium	SCI	-	Aneurysm clamping at the level of thoracic vertebrae	[40]
**Sandwich system: PLA fibers; NGF-loaded PLGA- microspheres-CS fibers**	Electrospinning (PLA fibers and CS fibers), ultrasonication and electrospraying (PLGA microspheres)	NGF	SCI	PC-12 cell line	Allen’s SCI models on rats	[41]
**CS tubular conduit**	Solvent casting in tubular mold and subsequent immersion in NaOH	FK506	PNI	-	Sciatic nerve injury rat model	[43]
**CS/glycerol tubular conduit**	Home-made tubular mold	Acetyl-L-carnitine	PNI	-	Left sciatic nerve transection on rats	[44]
**TMCSH-HC Nps**	Mixing of TMCSH and pDNA, lyophilization and addition of HC.	pDNA encoding for BDNF	PNI	-	Injection of nanoparticles before nerve crush injury induction	
**CS Nerve guide conduit with aligned microchannels loaded with halloysite nanotubes**	Unidirectional freezing in N_2_ and freeze-drying, cross-linking with epichlorohydrin	Aminopyridine	PNI	Schwann cell line	Sciatic nerve defect rat model	[22]
**PLLA nanotubes containing fibrin hydrogel loaded with curcumin encapsulated CS Nps and SCs**	Electrospinning (PLLA nanotubes)	Curcumin	PNI	Schwann cells	Sciatic nerve injury rat model	[46]
**CS/PLGA microspheres**	Re-emulsification TPP ionic cross-linking method	NGF	PNI	PC12 cells	Sciatic nerve injury rat model	[47]
**Associations**						
**Oxidized HA/glycol CS hydrogel**	Cross-linking and freeze-drying	tauroursodeoxycholic acid	SCI	-	Mechanical SCI rat model	[50]
**HA/CS injectable hydrogel**	Prepared at 37 °C, using ethyl-3-(3-dimethylaminopropyl) carbodiimide (EDC) and N-hydroxysuccinimide (NHS) and freeze-drying	NGF	PNI	BMMSCs	-	[48]
**RC-33 loaded-ALG nanofibers embedded in CS film**	Electrospinning (nanofibers), solvent casting (film)	RC-33	SCI	SH-SY5Y cells	-	[51]
**Berberine-loaded ALG/CS** **hydrogel**	CaCl_2_ cross-linked ALG added dropwise to CS solution containing β-glycerol phosphate	berberine	PNI	PC12 cells	crush-induced sciatic nerve rat model	[52]

**Table 2:** Summary of ALG-based DDSs for SCI and PNI application. DDS description, production technique, therapeutic agent delivered, in vitro and in vivo models exploited for efficacy assessment are reported.

DDS	Production Technique	Therapeutic Agent	Application	In Vitro Model	In Vivo Model	Reference
**PLA-based microfibers coated with a CaCl_2_- cross-linked ALG hydrogel layer**	Electrospinning (microfibers), cross-linking (hydrogel)	Rolipram	SCI	-	Rats subjected to C5 hemisection lesion	[55]
**CaCl_2_ cross-linked ALG- fibrinogen hydrogel embedded with PLGA microspheres**	Solvent extraction/evaporation (microspheres)	GDNF	SCI	PC-12 cells	Rat spinal cord hemisection model	[56]
**CaCl_2_ cross-linked ALG: fibrinogen-based hydrogel containing loaded CS-dextran sulfate Nps or PLGA microspheres**	CaCl_2_ cross-linking (hydrogel); not reported for microspheres and Nps	VEGF	SCI	SH-SY5Y and NIH-3T3 cells	Rat spinal cord hemisection model	[57]
**MH ALG or ALG-S complex; PLGA-based microspheres embedded in an ALG or ALG-S hydrogel**	Lyophilization for Hydrogel; single (oil/water) emulsion/solvent evaporation method for microspheres	MH and PCX	SCI	-	Left lateral hemisection animal (rats) model	[58]
**ALG-spermidine cross-linked hydrogel**	Ionotropic gelation and freeze-drying	Spermidine	PNI	Schwann cells	-	[59]

**Table 3:** Summary of Dx-based DDSs for SCI and PNI application. DDS description, production technique, therapeutic agent delivered, and in vitro and in vivo models exploited for efficacy assessment are reported.

DDS	Production Technique	Therapeutic Agent	Application	In Vitro Model	In Vivo Model	Reference
**Ibuprofen-Dx Nps**	Esterification between the hydroxyl groups of Dx and the carboxylic acid groups of ibuprofen, activated with N, N-carbonyldiimidazole.	Methylprednisolone	SCI	BV-12 microglial cells	Intraperitoneal injection in an SCI rat model	[62]
**Acetalated-Dx Nps**	Microprecipitation method	PCX	SCI	-	Mechanical SCI rat model	[63]
**Acetalated-Dx Nps nano-in-micro structured microspheres**	Microfluidic flow-focusing device	Methylprednisolone	SCI	-	Mechanical SCI rat model	[64]

**Table 4:** Summary of Dx-based DDSs for SCI. DDS description, production technique, therapeutic agent delivered, and in vitro and in vivo models exploited for efficacy assessment are reported.

DDS	Production Technique	Therapeutic Agent	Application	In Vitro Model	In Vivo Model	Reference
**Loaded-lipid microtubes embedded within AG injectable hydrogel**	Self-assembling of lipid microtubes, then added to AG solution.	BNDF	SCI	-	Dorsal over-hemisection rat model	[68]
**Loaded-lipid microtubes embedded within AG injectable hydrogel**	Self-assembling of lipid microtubes, then added to AG solution	BNDF, CA-Cdc42 and CA-Rac1	SCI	-	Modified dorsal-over hemisection rat model	[69]
**AG hydrogel containing loaded-PLGA Nps**	Double emulsion method (Nps)	PCX	SCI	-	Mechanical SCI rat model	[70]
**Loaded-lipid microtubes embedded in an AG-based hydrogel**	Thermal stabilization of chondroitinase ABC with trehalose	Chondroitinase ABC	SCI	-	Dorsal-over-hemisection injury	[71]
**AG scaffold with hexagonally packed multi-channel guides**	Multi-component fiber bundle templates (channels diameter: 166 μm)	Syngeneic marrow stromal cells expressing BDNF	SCI	-	Complete transection of rat severe injury model	[72]
**AG hydrogel dispersed with loaded PLGA Nps**	Nanoprecipitation method and lyophilization (Nps)	Estrogen (E2)	SCI	-	Moderate to severe SCI rat model	[73]
**Loaded-AG hydrogel**	AG prepared in artificial cerebrospinal fluid and then added with T3 as insoluble particles (obtained neutralizing)	Thyroid hormone 3,3′,5-triiodothyronine (T3).	SCI	-	Unilateral cervical spinal cord contusion injury rat model	[74]
**AG containing loaded-Dx sulfate complex**	Metal ion-assisted interaction	MH	SCI	-	Unilateral cervical spinal cord contusion injury rat model	[75]
**AG embedded with loaded- Dx-CS- particles**	Self-assembling by electrostatic interactions (particles)	BNDF	SCI	-	Unilateral cervical spinal cord contusion injury rat model	[76]
**AG scaffold with hexagonally packed multi-channel guides**	Multi-component fiber bundle templates (channels diameter of 200 μm)	BNDF	SCI	-	Sciatic nerve gaps rat model	[77]

**Table 5:** Summary of CL-based DDSs for SCI. DDS description, production technique, therapeutic agent delivered, and in vitro and in vivo models exploited for efficacy assessment are reported.

DDS	Production Technique	Therapeutic Agent	Application	In Vitro Model	In Vivo Model	Reference
**Biosynthesized cellulose (BC)** **tubes**	Custom-designed bioreactor with silicon tube as mold	NGF	SCI	PC12 cells	-	[81]
**CL-soy protein tubes seeded with SC cells**	Tubular mold	Pyrroloquinolinequinone (PQQ)	SCI	-	Sciatic nerve injury rat model	[82]

**Table 6:** Summary of GG-based DDSs for SCI. DDS description, production technique, therapeutic agent delivered, and in vitro and in vivo models exploited for efficacy assessment are reported.

DDS	Production Technique	Therapeutic Agent	Application	In Vitro Model	In Vivo Model	Reference
**CaCl_2_-cross-linked GG nanofibers embedded with loaded-GG freeze-dried matrix,**	Electrospinning (nanofibers), freeze-drying (porous matrix)	RC-33	SCI	-	-	[87]
**hydrogel conduits of GG and xanthan gum intercalated with polymethyl methacrylate particles**	Thermal-ionic cross-linking mechanism	Bovine serum albumin (BSA) and diclofenac sodium	PNI	-	-	[89]
**thiolated GG hydrogel**	Prepared at 60 °C and then poured into a mold to form in situ gel	Laminin and NGF	PNI	Rat neural stem cell	-	[90]
**GG/GL nanofibers**	Electrospinning	Spermidine	SCI/PNI	Schwann cells		[88]

## Data Availability

Not applicable.
